# A novel monoclonal IgG1 antibody specific for Galactose-alpha-1,3-galactose questions alpha-Gal epitope expression by bacteria

**DOI:** 10.3389/fimmu.2022.958952

**Published:** 2022-08-05

**Authors:** Luisa Kreft, Aloys Schepers, Miriam Hils, Kyra Swiontek, Andrew Flatley, Robert Janowski, Mohammadali Khan Mirzaei, Michael Dittmar, Neera Chakrapani, Mahesh S. Desai, Stefanie Eyerich, Li Deng, Dierk Niessing, Konrad Fischer, Regina Feederle, Simon Blank, Carsten B. Schmidt-Weber, Christiane Hilger, Tilo Biedermann, Caspar Ohnmacht

**Affiliations:** ^1^ Center of Allergy and Environment (ZAUM) and Institute of Allergy Research, Technical University of Munich, School of Medicine, and Helmholtz Center Munich, Research Center for Environmental Health, Neuherberg, Germany; ^2^ Monoclonal Antibody Core Facility, Helmholtz Center Munich, German Research Center for Environmental Health, Neuherberg, Germany; ^3^ Department of Dermatology and Allergy Biederstein, School of Medicine, Technical University of Munich, Munich, Germany; ^4^ Department of Infection and Immunity, Luxembourg Institute of Health (LIH), Esch-sur-Alzette, Luxembourg; ^5^ Institute of Structural Biology, Helmholtz Center Munich, German Research Center for Environmental Health, Neuherberg, Germany; ^6^ Institute of Virology, Technical University of Munich and Helmholtz Center Munich, German Research Center for Environmental Health, Neuherberg, Germany; ^7^ Chair of Prevention of Microbial Diseases, School of Life Sciences Weihenstephan, Technical University of Munich, Freising, Germany; ^8^ Odense Research Center for Anaphylaxis, Department of Dermatology and Allergy Center, Odense University Hospital, University of Southern Denmark, Odense, Denmark; ^9^ Chair of Livestock Biotechnology, School of Life Sciences Weihenstephan, Technical University of Munich, Freising, Germany; ^10^ German Center of Lung Research (DZL), Munich, Germany

**Keywords:** alpha-Gal, α-Gal, IgG, monoclonal antibody, carbohydrate, red meat allergy, xenotransplantation, bacteria

## Abstract

The alpha-Gal epitope (α-Gal) with the determining element galactose-α1,3-galactose can lead to clinically relevant allergic reactions and rejections in xenotransplantation. These immune reactions can develop because humans are devoid of this carbohydrate due to evolutionary loss of the enzyme α1,3-galactosyltransferase (GGTA1). In addition, up to 1% of human IgG antibodies are directed against α-Gal, but the stimulus for the induction of anti-α-Gal antibodies is still unclear. Commensal bacteria have been suggested as a causal factor for this induction as α-Gal binding tools such as lectins were found to stain cultivated bacteria isolated from the intestinal tract. Currently available tools for the detection of the definite α-Gal epitope, however, are cross-reactive, or have limited affinity and, hence, offer restricted possibilities for application. In this study, we describe a novel monoclonal IgG1 antibody (27H8) specific for the α-Gal epitope. The 27H8 antibody was generated by immunization of *Ggta1* knockout mice and displays a high affinity towards synthetic and naturally occurring α-Gal in various applications. Using this novel tool, we found that intestinal bacteria reported to be α-Gal positive cannot be stained with 27H8 questioning whether commensal bacteria express the native α-Gal epitope at all.

## 1. Introduction

Carbohydrates are vital and highly diverse structures that are variable between species. Of note, the alpha-Gal (α-Gal) epitope is a carbohydrate immunogen in humans that has relevance in allergy and xenotransplantation. The determining structure of the epitope is the disaccharide galactose-α1,3-galactose (Gal-α1,3-Gal), which naturally occurs as the trisaccharide galactose-α1,3-galactose-β1,4-N-acetylglucosamine (Gal-α1,3-Gal-β1,4-GlcNAc) on glycosylated proteins or lipids (1). The immunogenic property of the α-Gal epitope in humans is based on the loss of the enzyme α-1,3-galactosyltransferase (GGTA1) in Catarrhines, including apes and humans, which catalyzes the reaction of Gal-β1,4-GlcNAc−R + UDP-Gal to Gal-α1,3-Gal-β1,4-GlcNAc-R + UDP (2). Humans therefore do not express the α-Gal epitope in contrast to non-primate mammals. This absence eventually allows for the sensitization of humans and a subsequent development of the so-called ‘α-Gal syndrome’ or red meat allergy that is based on the formation of IgE molecules against α-Gal *via* tick bites (3–5). These IgE molecules may lead to allergic reactions including fatal anaphylaxis following ingestion of mammalian meat or related products such as gelatin or innards, for instance pork kidney, which are major sources of allergen in α-Gal-induced meat allergy (6–9). Moreover, sensitization to α-Gal can also result in severe allergic reactions in cancer patients who receive Cetuximab, a chimeric human-murine monoclonal antibody that contains α-Gal on the Fab fragment (10). Interestingly, antibodies of different isotypes against the α-Gal epitope are quite abundant in humans with IgG levels estimated to range between 1% (11) to 0.1% of total plasma IgG with high variability between subjects and lowest abundance in individuals carrying the blood type B antigen (12). The latter observation is likely due to the structural similarity between the α-Gal epitope and blood type B antigen, which contains an additional fucose molecule on the second last galactose molecule (1). These human anti-α-Gal antibodies pose a challenge for xenotransplantation, in particular for pig organ transplantation, which was overcome to some extend with developing *GGTA1* knockout (KO) pigs (13–15). The induction of anti-α-Gal antibodies has been hypothesized to be mediated by the gut microbiota, since intestinal bacteria are recognized by anti-α-Gal binding molecules, such as purified polyclonal human anti-α-Gal antibodies (16, 17) or Isolectin B4 from *Bandeiraea simplicifolia* (BSI-B_4_) (18–20). Furthermore, antibiotics have shown to reduce preexisting anti-α-Gal antibodies of the IgG isotype (21) and the oral introduction of *Escherichia coli* O86:B7 in *Ggta1* KO mice has been shown to induce anti-α-Gal antibodies (IgG, IgM) (22). BSI-B_4_ and another α-Gal-binding lectin from the mushroom *Marasmius oreades* (MOA) (23) have reduced specificity to the α-Gal epitope, as they both also bind to the blood group B antigen. The currently most widely used α-Gal-specific monoclonal antibody is M86, an IgM antibody which was developed by Galili *et al.* in *Ggta1* KO mice (24) and to some degree also chicken single-chain antibody variable-region fragments (scFv) against α-Gal developed by Cunningham *et al.* (25). Neither of the two antibodies has been convincingly shown to stain bacteria to the authors’ knowledge. However, the monoclonal antibody M86 was indeed used to show α-Gal expression on parasites such as *Plasmodium* species (18). As the M86 antibody is of the IgM isotype with limited affinity and purification properties, we aimed to establish a novel IgG antibody with high affinity for both the di- and trisaccharide α-Gal epitope and with wide applicability.

Here, we report the development of a novel IgG1 antibody called 27H8 that is highly specific for both synthetic and naturally occurring α-Gal epitopes. The 27H8 monoclonal antibody shows high affinity to the α-Gal epitope and offers wide applicability for α-Gal detection such as in ELISA, dot blots, immunohistochemistry and flow cytometry. Using the 27H8 antibody, we did not find any specific binding to bacteria originating from the intestinal tract while cross-specific BSI-B_4_ readily stained cultured or intestinal bacteria. Altogether, our newly developed antibody can be used as a novel tool for α-Gal detection with high sensitivity and specificity. Lastly, our results question the role of the intestinal microbiota as a major source of the α-Gal epitope for sensitization.

## 2. Material and methods

### 2.1. Animal ethics statement

*Ggta1* KO and wildtype (WT) mice were kept under specific pathogen-free conditions. All interventions were performed in accordance with the European Convention for Animal Care and Use of Laboratory Animals and were approved by the local ethics committee and appropriate government authorities (ROB-55.2-2532.Vet_03-17-68). *GGTA1* KO pigs were developed and maintained according to ROB-55.2-2532.Vet_02-18-56.

### 2.2. Patient and control sera

Serum samples were retrieved from atopic dermatitis patients (male n=13, female n= 6) with a mean SCORAD of 63 ± 14.2, α-Gal allergic patients with α-Gal-syndrome confirmed by α-Gal specific IgE, medical history or oral provocation test (n=7) or healthy controls (n=17). All individuals gave written consent and the study collection was approved by the local ethics committee. The α-Gal allergic patients and healthy controls were part of the BioBank of the Department of Dermatology and Allergy Biederstein, School of Medicine, Technical University of Munich, Munich, Germany and approved by ethical vote 419/18 S-KK. The atopic dermatitis patient collection was approved by ethical vote 5590/12.

### 2.3. Immunization protocol and hybridoma generation

α1,3-galactosyltransferase 1 (*Ggta1*) knock-out (KO) mice (26) kindly provided by the group of Florian Kreppel, University of Ulm, Germany were immunized subcutaneously (s.c.) and intraperitoneally (i.p.) with a mixture of 50 µg ovalbumin-coupled Gal-α1,3-Gal-β1,4-GlcNAc trisaccharide (α-Gal-OVA, 14-atom spacer, Dextra, Reading, UK) in 200 µl PBS, 5 nmol CpG2006 (TIB MOLBIOL, Berlin, Germany), and 200 µl Incomplete Freund’s adjuvant (Sigma-Aldrich, St. Louis, MO, USA). After 11 weeks, a boost without Freund’s adjuvant was given i.p. and s.c. 3 days before hybridoma fusion. Fusion of the myeloma cell line P3X63-Ag8.653 with mouse splenic B cells was performed using polyethylene glycol 1500 according to standard procedure (27). After fusion, hybridoma cells were plated in 96-well plates using RPMI 1640 supplemented with 15% fetal calf serum, 1% glutamine, 1% pyruvate, 1% non-essential amino acids and 2% HAT media supplement (Hybri-Max, Sigma-Aldrich). Hybridoma supernatants were screened 10 days later in a flow cytometry assay (iQue, Intellicyt; Sartorius, Göttingen, Germany) using BSA-coupled Gal-α1,3-Gal (α-Gal-DI-BSA, 3-atom spacer, Dextra) captured on 3D-aldehyde beads (PolyAN, Berlin, Germany). Beads were incubated for 90 minutes (min) with hybridoma supernatant and Atto-488-coupled isotype-specific monoclonal rat anti-mouse IgG secondary antibodies. Antibody binding was analyzed using ForeCyt software (Sartorius). Positive supernatants were further validated by dot blot and cells from clone 27H8 were sub-cloned by five rounds of limiting dilution to obtain stable monoclonal hybridoma cell lines (mouse IgG1/ƙ).

### 2.4. Purification of the 27H8 antibody

Hybridoma supernatant from subcloned 27H8 was purified on an ÄKTA Pure chromatography system (Cytiva) using Cytiva HiTrap Protein A HP column (Fisher Scientific, Waltham, MA, USA).

### 2.5. Screening material

Mouse serum albumin (MSA) was purchased from Sigma-Aldrich, Ovalbumin (OVA) EndoFit from *In vivo*Gen, San Diego, CA, USA and Bovine Serum Albumin (BSA) from AppliChem, Darmstadt, Germany (Albumin Fraction V). Further proteins coupled to the α-Gal epitope Gal-α1,3-Gal-β1,4-GlcNAc (referred to as “TRI”-saccharide), Gal-α1,3-Gal-β1,4-GlcNAc-MSA (α-Gal-MSA, 3 atom spacer) and Gal-α1,3-Gal-β1,4-GlcNAc-BSA (α-Gal-TRI-BSA, 3 atom spacer) were purchased from Dextra. α-Gal-rich glycolipids were extracted from rabbit erythrocytes (Innovative Research, Novi, MI, USA) as described previously (28), modified from (29, 30). Bovine thyroglobulin was purchased from Merck, Darmstadt, Germany. His-tagged porcine aminopeptidase N (APN) was recombinantly produced in human embryonic kidney (HEK) 293 cells (APN control without α-Gal) (28) as well as in HEK293 cells stably expressing murine GGTA1 (α-Gal-APN) (31). The cells were cultured in DMEM supplemented with 10% FCS, penicillin and streptomycin. After reaching 70% confluency, FCS-containing medium was removed and cells were gently washed once with PBS. Fresh DMEM (Sigma-Aldrich) containing PeproGrow-1 (serum-free cell culture supplement, PeproTech) was added and cells were cultured for further 4 - 6 days without medium exchange until cell viability showed the first signs of deterioration. Medium supernatant was harvested and passed through a 0.45 µm (Sarstedt, Nürnberg, Germany) filter to remove residual cell debris. The recombinant proteins were purified from the filtrate using Ni-NTA affinity chromatography and subsequent gradient elution with imidazole (AppliChem). Protein-containing fractions were screened for purity *via* SDS-PAGE and subsequent staining with Coomassie blue. Suitable fractions were pooled. Proteins were concentrated using centrifugal filter units (Amicon Ultra-15, Merck), including a final washing step with PBS to reduce the imidazole concentration to ≤ 20 mM. After sterile filtration (Millex-GV Syringe 0.22 µm Filter Unit, Merck) and shock freezing in liquid N2, proteins were stored at -80°C until usage.

### 2.6. Screening lysates

Pig wildtype (WT) kidney was derived from a local butcher. Pig KO kidney samples were derived from *GGTA1*-gene knockout (KO) pigs (32). Cultivated *GGTA1* KO and WT pig kidney cells were lysed with Cytobuster (Merck). 0.5 cm x 0.25 cm tissue pieces of pig kidneys (WT/KO) were lysed in 1 ml RIPA buffer containing 50 mM Tris buffer, pH 8.0 (AppliChem), 150 mM sodium chloride (AppliChem), 1% Nonidet P-40 (AppliChem), 0.5% sodium deoxycholate (Sigma-Aldrich) and 0.1% sodium dodecyl sulfate (Sigma-Aldrich). 10 ml RIPA buffer contained 1 tablet Protease Inhibitor Cocktail (Roche, Basel, Switzerland) and 1 tablet PhosSTOP (Roche). Tissue samples were homogenized using metal balls in a TissueLyser LT (Quiagen, Hilden, Germany) at 50 Hz 3 min, sonicated for 10 seconds and centrifuged at 16,000xg for 30 min at 4°C. Protein amounts in the collected supernatants were measured with a Pierce BCA Protein Assay Kit (Thermo Fisher Scientific) using bovine serum albumin (BSA) as standard.

500 µl of whole blood from a donor with blood group B was centrifuged at 2000xg for 10 min and the cell pellet frozen at -80°C before adding 1 ml RIPA buffer containing protease and phosphatase inhibitor as described above. Cells were sheared by massive pipetting and vortexing steps and then incubated on ice for 30 min before centrifuging at 16,000xg for 30 min at 4°C. The supernatant was collected and stored at -80°C until usage.

### 2.7. Bacterial strains and lysates

*Staphylococcus aureus* strains Mu50, SA113, COL, 20231, RN1, SH1000, MW2, RN4220, Newman, USA300, *Escherichia coli* strains (K12, DH5α), *Helicobacter pylori* (J99), *Pseudomonas aeruginosa* (DSM 50071), *Haemophilus influenza* (Hi375), *Acinetobacter baumannii* (ATCC 17978), *Salmonella typhimurium* (ATCC 14028) were purchased from ATCC Manassas, VA, USA and DSMZ, Leibniz Institute, Germany. *Akkermansia muciniphila* was obtained from Willem De Vos at Wageningen University. The bacteria were grown overnight at 37°C to a density of 10^9^ CFU/ml. All bacteria were grown in Luria Bertani (L.B.) broth (tryptone 10g, NaCl 10g, yeast extract 5g in 1L H_2_O, adjust pH to 7.0 with 5 N NaOH, sterilize), except for *H. pylori* in Brain Heart Infusion (BHI) (beef heart, 5 g/L, calf brains, 12.5 g/L, disodium hydrogen phosphate, 2.5 g/L, D(+)-glucose, 2 g/L, peptone, 10 g/L, sodium chloride, 5 g/L) plus 20% fetal calf serum (FCS), *H. influenza* in BHI 37g, NAD 15mg, and Hemine 15mg in 1L H_2_O, and *Akkermansia muciniphila* (ATCC BAA-835) in reduced BHI. Pelleted bacteria (approximately 3x10^9^ bacterial cells) were washed with PBS and resuspended in 1 ml RIPA buffer as described for mammalian samples, and added to glass beads and beat for one hour (max speed 2800 rpm using a Vortex shaker) and transferred to new tubes for storage at -80°C.

*E. coli* HS was originally isolated from a human fecal sample of a healthy adult (33). *E. coli* O86:B7 and *Lactobacillus rhamnosus* were purchased from the American Type Culture Collection (ATCC 12701 and 53103), *E. coli* BL21 from Thermo Fisher Scientific (EC0114). *E. coli* strains were grown overnight at 37°C in LB medium, *L. rhamnosus* was grown overnight at 37°C in Lactobacilli MRS broth (proteose peptone #3 10 g, beef extract 10g, yeast extract 5g, dextrose 20g, sorbitan monooleate 1g, ammonium citrate 2g, sodium acetate 5g, MnSO_4_ x H_2_O 0.05g, Na_2_HPO_4_ 2g in 1L H_2_O, adjust pH to 6.5). RIPA buffer was added to cell pellet of 5 ml culture and cells were lysed for 30min at 30Hz with glass beads.

### 2.8. Enzymatic digestion and cleavage of the α-Gal epitope

Glycolipids were digested by Endoglycoceramidase I (EGCase I) using a ratio of 1 µg Glycolipids per 1 milliunit enzyme in 1x EGCase I Reaction buffer (New England Biolabs, MA, USA) in PBS for 37°C for 16 hours. Precipitated enzyme was removed after heat inactivation for 20 min at 65°C. 2 µg/ml pig kidney tissue lysates were digested with α-Galactosidase from green coffee beans (Sigma Aldrich) at 10 U/ml in 100 mM potassium phosphate buffer, pH 6.5 for 3 hours at room temperature (RT). Ammonium sulfate was removed from α-Galactosidase preparation before digest by pelleting the enzyme through a centrifugation step at 15,000xg for 10 min at 4°C. The supernatant was collected and the pellet resuspended in an equal volume of potassium phosphate buffer. *S. aureus* lysate was digested by adding 5 µl of whole lysate to 5 µl potassium phosphate buffer containing 20 U/ml α-Galactosidase (end concentration 10 U/ml) and further processed as described before. For EGCase I digestion, 10 µl bacterial lysate was digested in 1x EGCase I reaction buffer diluted with PBS and 1 µl EGCase I as described above (end volume 20 µl).

### 2.9. Dot blot screening approach

**Figure 1:**
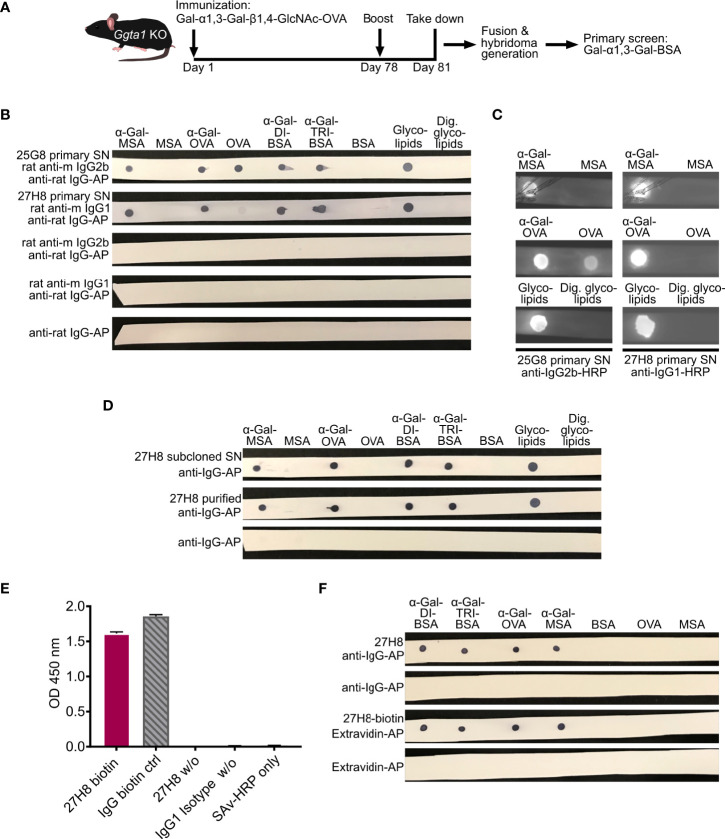
Generation, screening and biotinylation of a monoclonal IgG antibody recognizing galactose-α1,3-galactose. **(A)** Schematic approach for the generation of a monoclonal anti-α-Gal antibody through immunization of *Ggta1* KO mice with Gal-α1,3-Gal-β1,4-GlcNAc-OVA (α-Gal-OVA) and screening of primary hybridoma supernatants (SNs) with Gal-α1,3-Gal-BSA (α-Gal-DI-BSA). **(B)** Dot blot of 25G8 and 27H8 primary SNs on α-Gal-conjugated glycoproteins or -lipids and respective negative control proteins devoid of α-Gal. Endoglycoceramidase I (EGCase I)-digested glycolipids (right) served as negative control for glycolipids. Unlabeled rat anti-mouse (anti-m) isotype-specific secondary antibodies and anti-rat-tertiary antibody labeled with alkaline phosphatase (AP) were used for detection. **(C)** Secondary screen on dot blots of 25G8 and 27H8 primary hybridoma SNs on α-Gal carrying glycoproteins and the respective negative control proteins. Detection was performed with HRP-labeled secondary antibodies. See **Supplementary Figure 1A** for uncropped blots. **(D)** 27H8 subcloned hybridoma SN and 27H8 purified antibody were screened as in **(B)**. **(E)** ELISA of 27H8 biotinylated antibody (27H8-biotin), IgG-biotin control, non-biotinylated 27H8 (27H8 w/o) and IgG1 Isotype control (IgG1 Isotype w/o) coated onto plates and detected by Streptavidin-HRP. For details, see Material and Methods section. **(F)** Biotinylated 27H8 antibody detected with Extravidin-AP was compared to unlabeled purified 27H8 antibody (w/o) detected by anti-IgG-AP. Further abbreviations **(A-F)**: Ggta1, α-galactosyltransferase; KO, knockout; OVA, Ovalbumin; BSA, bovine serum albumin; Ig, Immunoglobulin; MSA, mouse serum albumin; Dig., digested; w/o, without; HRP, horseradish peroxidase; ctrl, control; SAv, streptavidin.

**Figure 2:**
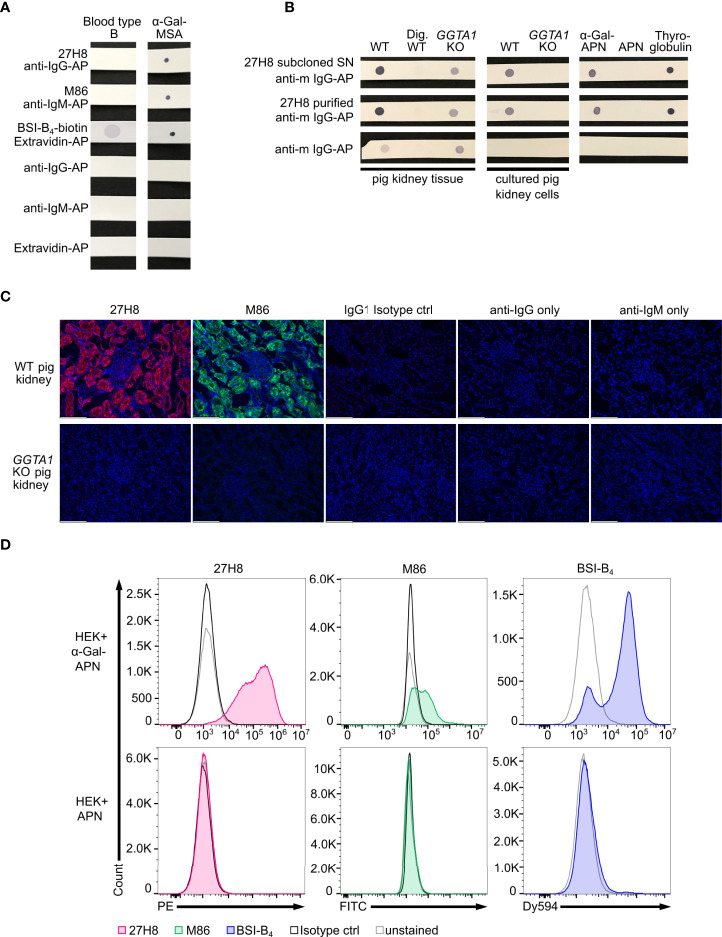
Specificity of 27H8 monoclonal antibody. **(A)** Dot blot stain of lysed whole blood from a type B blood donor and α-Gal-MSA (positive control) by 27H8, M86 or Lectin (BSI-B_4_). **(B)** Screening of 27H8 subcloned hybridoma SN (upper row) and purified 27H8 antibody (middle row) on lysed kidney tissue or cultured kidney cells of wildtype (WT) and *GGTA1* knockout (KO) pigs and on WT kidney tissue samples digested with α-Galactosidase (Dig. WT). Further screening molecules: aminopeptidase N (APN) glycosylated with α-Gal, APN only and (α-Gal-containing) bovine thyroglobulin. **(A, B)** Samples in a row were blotted on one membrane. See **Supplementary Figure 1** for uncropped blots. **(C)** Immunofluorescence microscopy of pig kidney tissue specimens (WT and *GGTA1* KO) stained with 27H8 (red) and M86 (green) in the glomerulus region. IgG1 isotype ctrl and secondary antibody only stains (anti-IgG1/anti-IgM) served as controls for fluorescence signal. DNA stained with DAPI (blue). Scale bar (white, left corner): 124.4 µm. **(D)** Flow cytometry analysis of human embryonic kidney (HEK) cells expressing α-Gal glycosylated APN (upper panel) and APN only (lower panel) stained with 27H8 (red), M86 (green) and BSI-B_4_ (blue). Controls: unstained (grey) and Isotype controls (IgG1, IgM, both in black). **(A–D)** If not otherwise indicated, 27H8 was applied in the purified version.

Nitrocellulose membranes (Carl Roth, Karlsruhe, Germany) were cut into length of 10 cm x 0.5 cm and 1 µl of sample was applied 1 cm apart to a maximum of 10 samples per membrane strip, except for horseradish peroxidase (HRP) detection for which 2 µl were spotted (**Figure 1C**). The amount of blotted α-Gal conjugated glycoproteins and proteins devoid of α-Gal was 0.1 µg (**Figures 1E**, **4A, F**, **5B**), 1 µg (**Figures 1B, D**, **2A**) or 2 µg (**Figure 1C**) per dot. 0.125 µg of glycolipids (with or without EGCase I digestion) and 1 µl of the whole blood lysate from a blood type B donor were spotted per dot. Pig kidney and cell lysates of cultured pig cells (pre-digested or not), α-Gal-APN, APN and thyroglobulin were spotted at an amount of 1 µg. Whole bacterial lysates were spotted at 1 µl without protein amount normalization. After a drying time of 15 min, the membrane was transferred to a chamber of mini-incubation trays (Bio-Rad Laboratories, Hercules, CA, USA) and blocked with 1.5 ml 2% BSA (Albumin Fraction V, AppliChem) in Tris-buffered saline (TBS, 20 mM Tris, 150 mM NaCl, pH 7.6, both AppliChem) for 1 hour at RT. Primary antibodies and lectin were incubated over night at 4°C and diluted in 1 ml TBS supplemented with 1% BSA. Primary hybridoma supernatants from clones 27H8 and 25G8 were used at a 1:5 dilution in, purified and biotinylated 27H8 antibody at 0.6 µg/ml, M86 hybridoma supernatant (Enzo Life Sciences Farmingdale, NY, USA) in a 1:5 dilution and biotinylated lectin from *Bandeiraea simplicifolia* (BSI-B_4_, Sigma-Aldrich) at 25 µg/ml. IgG isotype control (Invitrogen, Carlsbad, USA; polyclonal, **Figure 4**) and IgG1 isotype control (Clone P3.4.2.8.1., Thermo Fisher Scientific, **Figure 5B**) for bacterial samples were used at 0.6 µg/ml. After primary detection, membranes were washed three times for 5 min with 1 ml 0.05% Tween20 (Calbiochem, Merck) in TBS (TBS/T). Secondary detection antibodies were incubated for 90 min at RT in 1 ml TBS/1%BSA. 27H8 primary hybridoma supernatant was detected by a monoclonal rat anti-mouse IgG1 (2E6, in house), 25G8 primary hybridoma supernatant was detected by a monoclonal rat anti-mouse IgG2b (7B8, in house) and in a tertiary incubation step with alkaline phosphatase (AP)-conjugated anti-rat IgG with minimal cross-reactivity (Jackson ImmunoResearch, Philadelphia, PA, USA) at a dilution of 1:5000. Both, supernatant from subcloned 27H8 and purified 27H8 antibody was detected by AP-conjugated anti-mouse IgG, Fc-specific (Sigma-Aldrich) at a dilution of 1:10,000. M86 was detected by AP-conjugated μ-chain specific anti-mouse IgM (Sigma-Aldrich) at a dilution of 1:30,000; BSI-B_4_ and biotinylated 27H8 by AP-conjugated Extravidin (Sigma-Aldrich) at 1:10,000. Membranes were washed three times in TBS/T for 5 min and immersed in 0.01% nitro blue tetrazolium (AppliChem) and 0.005% 5-bromo-4-chloro-3-indolyl phosphate (AppliChem) in detection buffer (100mM Tris, 10 mM MgCl_2_*6H_2_O, 100 mM NaCl, pH 9.5) until dots were stained. After immersing membranes with distilled water, membranes were dried, aligned on a black paper and acquired with a photo camera at 15 cm height. For direct detection with horseradish peroxidase (HRP)-labeled secondary antibodies, glyco-/proteins or glycolipids were spotted on a membrane strip pre-wet in transfer buffer containing 25 mM Tris, 19.2 mM glycin and 20% isopropanol, pH 8.3. Rat anti-mouse-IgG1 (2E6, in house) labeled with HRP (for 27H8 primary supernatant) and rat anti-mouse-anti-IgG2b-HRP (for 25G8 primary supernatant). Uncropped dot blots from all figures are displayed in **Supplementary Figure 1**.

### 2.10. Periodic acid treatment

Nitrocellulose membranes with blotted samples were incubated in 40 mM periodic acid (H_5_IO_4_, Merck) diluted from a stock concentration of 200 mM in 50 mM sodium acetate buffer (AppliChem, adjust to pH 4.5 with HCl) for 1 hour at RT.

### 2.11. Antibody biotinylation

Purified 27H8 antibody was labeled with biotin-7-NHS using a Biotin Protein Labeling Kit (Roche) at a molar ratio of 1:10. Excess biotin-7-NHS was removed by gel filtration according to manufacturer’s instructions. To verify the biotinylation efficiency, biotinylated 27H8 and a random biotinylated IgG antibody (biotinylated rat anti-mouse IgM, clone R6-60.2, BD, Franklin Lakes, USA) and as controls 27H8 without biotinylation and IgG1 isotype control (Southern Biotech, Birmingham, AL, USA) were coated at 2 µg/ml on a flat bottom MaxiSorp 454 96-well plate (Thermo Fisher Scientific) in 50 µl/well in sodium carbonate-bicarbonate buffer (pH 9.5) for 16 hours. The plate was washed three times with PBS/T (0.05% Tween), blocked with 300 µl 1% BSA and washed again three times. 50 µl streptavidin-HRP (BD) diluted 1:250 was added for 1 hour. Plate was washed again eight times and 50 µl 3,3′,5,5′-tetramethylbenzidin (1-Step Ultra TMB ELISA, Thermo Fisher Scientific) was added. Reaction was stopped with 25 µl 2M H_2_O_2_ (Merck) and emission was measured at 450 nm using a plate reader (Epoch, Biotek, Thermo Fisher Scientific).

### 2.12. Immunohistochemistry

0.25 cm x 1 cm sections of WT and *GGTA1* KO pig kidneys were fixed in 3.6% buffered formaldehyde (Fischar, Saarbrücken, Germany) for 24 hours and embedded in paraffin. Sections of 4 µm were cut and transferred to slides. Slides were washed twice with Xylol (Carl Roth, Karlsruhe, Germany) for 10 min. For rehydration, slides were transferred into a graded series of ethanol in distilled water: 100% (twice, 5 min), 96% (5 min), 70% (5 min), 50% (1 min), H_2_O (30 seconds) and washed for 5 min in PBS (Sigma-Aldrich). Antigen was retrieved by transferring slides into nearly boiling citrate buffer, incubating at 90°C (10 min) and slowly cooling to RT (~30 min). Slides were washed 5 min in PBS and blocked with 2% BSA in PBS for 1 hour. Tissue sections were incubated with primary antibody solutions in 1% BSA at the following concentrations/dilutions: 1 µg/ml 27H8 or IgG1κ isotype control (Clone: P3.6.2.8.1, unconjugated, eBioscience) or 1:5 dilution of M86 supernatant (IgM) for 16 hours at 4°C. Slides were washed three times with PBS for 5 min and incubated for 30 min at RT with fluorochrome labeled secondary antibody diluted in 1%BSA in PBS at the following concentrations: goat anti-mouse IgG (H+L) conjugated to Alexa Fluor 647 (polyclonal, Thermo Fisher Scientific) at 2 µg/ml and goat anti-mouse IgM (heavy chain) conjugated to Alexa Fluor 488 (polyclonal, Thermo Fisher Scientific) at 10 µg/ml. Slides were washed in PBS (three times, 5 min) and 1 drop of ProLong Diamond Antifade mounting medium with DAPI (Life Technologies by Thermo Fisher) was added. Images were acquired on a Leica DM4B fluorescence microscope and processed using LAS X software (Leica, Wetzlar, Germany) with a 20X objective. Contrast and brightness were adjusted simultaneously on all images per channel with ImageJ software (https://imagej.nih.gov/ij/, Rasband, W.S., U. S. National Institutes of Health, Bethesda, MD, USA). All antibody solutions were centrifuged to remove antibody complexes before use.

### 2.13. Eukaryotic flow cytometry

HEK cells expressing α-Gal glycosylated APN and APN devoid of α-Gal (see description in screening material above) were washed with BSA-Buffer containing 1% BSA in PBS. 5x10^5^ cells were seeded and stained with either 27H8 purified antibody or IgG1κ isotype control (clone: B3102E8, Southern Biotech) at 1 µg/ml, a 1:10 dilution of M86 supernatant, a 1:10 dilution (40 µg/ml) of mouse IgM isotype control (clone: MOPC 104E, Sigma) or a 1:100 dilution of BSI-B_4_ conjugated to DyLight^®^594 (*Griffonia simplicifolia* isolectin B4, Vector Laboratories, Burlingame, CA, USA) in BSA-buffer. Cells were washed twice and stained with respective secondary antibodies: anti-mouse IgG1-PE (clone: A85-1, BD Pharmingen) at a 1:100 dilution or anti-mouse IgM-Alexa Fluor^®^ 488 (clone: 1B4B1, Southern Biotech) at a 1:500 dilution. Staining was performed in 100 µl for both primary and secondary antibody incubation steps for 45 min. Cells were washed twice and resuspended in 150 µl BSA-buffer before acquisition at a Novocyte Quanteon Flow Cytometer.

For murine splenocyte staining, spleen was excised and meshed on a 70 µM filter for generating a single cell suspension. After washing splenocytes twice with PBS, erythrocytes were lysed with an ACK lysis buffer (155 mM NH_4_, 10 mM KHCO_3_, 0.1 mM EDTA-2Na-2H_2_O; pH 7.2-7.4) in 1 ml for 2 min. Cells were washed with BSA-SA-buffer twice before staining. Staining and acquisition were performed as described for intestinal bacterial staining (see protocol below), except that splenocytes were stained with 5 µg/ml 7-Aminoactinomycin D (7AAD, Enzo Life Sciences) instead of SYBR green identification of living cells (**Supplementary Figure 2B**).

### 2.14. Bacterial flow cytometry

Bacteria were grown to a density of 10^9^ CFU/ml. *S. aureus* 20231 and *E.coli* K12 were grown overnight at 37°C in L.B. medium. 100 µl of the culture was seeded into a 96-well U-bottom plate and washed with BSA-SA-buffer containing 1% BSA, 0.05% sodium azide (Morphisto, Offenbach am Main, Germany) in PBS at 4000xg for 5 min. Cells were stained with 1 µg/ml 27H8 and washed twice and stained with anti-IgG1-PE antibody (clone A85-1, BD, Franklin Lakes, NJ, USA) at a 1:100 dilution in a total volume of 50 µl. Bacteria were washed twice and fixed for 30 min with 3.7% formaldehyde (AppliChem, 37% diluted 1:10 in PBS) and washed again twice before acquisition in 100 µl PBS at an Acurri™ Flow Cytometer (BD). *E. coli* O86:B7, BL21, HS and *L. rhamnosus* were cultivated o.n. at 37°C in 5 ml L.B. medium shaking at 150 rpm. Cells were centrifuged (4000xg, 5 min) and washed twice with PBS before fixing cells in 4% PFA for 30 min. Bacteria were washed with PBS and then stained with primary and secondary antibody as described for HEK cells (see protocol above). Bacterial pellet was resuspended in 100 µl BSA-buffer for acquisition on a Novocyte Quanteon Flow Cytometer. In general, at least 5 x 10^5^ events were acquired.

For intestinal bacteria staining of *Ggta1* KO and WT mice, the entire small intestine, cecum and colon were removed. Small intestine was cut longitudinally and whole content streaked out with a sterile pipette tip into a 1.5 ml tube. The cecum was cut on the tip and 2/3rds of the content streaked out. For the colon, the whole content was streaked out. 1 ml BSA-SA-buffer was added and slurry mixed by vortexing and pipetting. Intestinal debris was spun down at 900xg for 5 min, 4°C, and supernatant was transferred to a new tube for another centrifugation step at 450xg for 5 min, 4°C, to remove host cells. Bacterial pellets were washed twice in 1 ml BSA-SA-buffer at 8000xg 5 min, 4°C and filtered (70 µM) before seeding 100 µl of washed small intestine content, 25 µl of cecum content, 50 µl of colon content into U-bottom plates. Pellets were centrifuged, supernatant removed and stained in 50 µl for 30 min. Concentrations and dilutions were: 1 µg/ml biotinylated 27H8 or IgG1κ isotype control (clone P3.6.2.8.1., biotinylated, eBioscience/Thermo Fisher Scientific) or 1:40 dilution of biotinylated BSI-B_4_. Before staining with a 1:500 dilution of streptavidin-PE (SAv-PE, BD) cells were washed twice by a centrifugation step at 3200xg for 5 min at 4°C. After two additional washing steps, bacteria were resuspended in 200 µl of a 1:100,000 dilution of SYBR green (SYBR green I nucleic acid gel stain, Sigma-Aldrich), incubated for 5 min and acquired at an Acurri™ Flow Cytometer. Data analysis of FCS-files was performed with FlowJo (Version 10.7.1) and SYBR green positive were considered as bacteria (**Supplementary Figure 2A**) as described in (34).

### 2.15. Surface Plasmon Resonance analysis

The binding measurements were performed on a BIACORE 3000 instrument (Biacore Inc., Piscataway, NJ, USA) and analyzed with Origin software version 9.0. 27H8 purified antibody was diluted to a final concentration of 50 nM in 10 mM sodium acetate, pH 4.0, and chemically immobilized (amine coupling, 850 RU bound) onto CM5 sensor chip (Cytiva). α-Gal-DI-BSA and α-Gal-TRI-BSA were diluted in the running buffer (PBS, 1 mM DTT and 0.005% Tween 20) to the final concentration of 0.977 nM, 1.95 nM, 3.91 nM, 7.81 nM, 15.6 nM, 31.3 nM, 62.5 nM, 125 nM, 250 nM, 500 nM and injected over the sensor chip surface at 30 µL/min at 25°C. The protein samples were injected onto the sensor chip from the lowest to the highest concentration. Both glycoprotein samples were tested three times. Injection of 250 nM ligand was performed in duplicate within each experiment. In order to subtract background noise from each experiment, all samples were run over an unmodified CM5 sensor chip surface. After each ligand injection, the sensor chip was regenerated using 3 M MgCl_2_ solution. For each measurement the equilibrium dissociation constant was calculated (K_D_). The K_D_s from three experiments were used to calculate the mean values of these variables and the standard deviation.

### 2.16. Enzyme-linked Immunosorbent Assay

For comparing supernatant from subcloned 27H8 hybridoma and M86 hybridoma supernatant, both antibodies were titrated on glycoproteins coated to standard ELISA plates. α-Gal-DI-BSA, α-Gal-TRI-BSA, α-Gal-OVA, α-Gal-MSA and respective negative control proteins BSA, OVA and MSA were coated at a concentration of 5 µg/ml in 50 µl per well on a flat bottom Maxi-Sorp 96-well plate (Thermo Fisher Scientific) for 12 hours at 4°C in sodium carbonate-bicarbonate buffer, pH 9.5. Plates were washed three times with PBS/T and blocked with BSA-buffer for 1 hour at RT and washed again three times with PBS/T. 27H8 supernatant and M86 were titrated in BSA-buffer starting from 1.12 µg/ml in a serial 1:10 dilution to 1.12 ng/ml. The starting concentration was set according to the stock concentration of the M86 antibody in the hybridoma supernatant. The amount of 27H8 and M86 antibody in the hybridoma supernatants was measured with a Biotech Clonotyping System-HRP Kit and mouse Immunoglobulin Panel for Standards (both Southern Biotech) according to the manufacturer’s instruction, yielding a concentration of 116.69 µg/ml 27H8 antibody in the supernatant and 1.12 µg/ml of M86. IgG1 and IgM Isotype controls (Southern Biotech) were used at the highest concentration at 1.12 µg/ml. Primary antibodies incubated for 1 h at RT and plates were washed 5x with PBS/T. Polyclonal antibody conjugated to horseradish peroxidase (HRP) detecting both mouse IgG and IgM heavy and light chains (Jackson ImmunoResearch, West Grove, PA, USA) were incubated at a concentration of 80 pg/ml (1:10,000 dilution) in 1% BSA in PBS in 50 µl per well for 1 hour at RT shaking at 450 rpm. Plates were washed again 8 times with PBS/T and 50 µl TMB substrate (1-step Ultra TMB, Thermo Fisher Scientific) was added before stopping the reaction with 25 µl 2M sulfuric acid (Merck). Emission was measured with a plate reader at 450 nm. ELISAs were repeated three times. Analysis, logarithmic transformation and curve fit (nonlinear variable slope, 4 parameters) was performed with GraphPad Prism 7 (GraphPad Software Inc.)

For epitope blocking ELISAs, α-Gal MSA was coated onto plates at 0.5 µg/ml in 50 µl per well as described before. Plates were washed with PBS/T, blocked with BSA-buffer and washed again as described before. Blocking antibody 27H8 supernatant was added in a serial dilution (1:10) from 100 µg/ml to 0.01 µg/ml in BSA-buffer. As the concentration of M86 was low compared to 27H8 in supernatant, a serial dilution of M86 was applied from 1 µg/ml to 0.01 µg/ml. The blocking antibody was incubated for 1 hour at RT with shaking at 500 rpm and plates were washed 5 times. Afterwards, the competing antibody (27H8 supernatant for M86 block and M86 for 27H8 supernatant block) was incubated for 1 hour at a concentration of 0.1 µg/ml at RT and shaking at 500 rpm and wells were washed 5 times. Detection was performed with either anti-IgG1-HRP for 27H8 competing antibody or anti-IgM-HRP for M86 competing antibody (both from Southern Biotech) at a 1:500 dilution. TMB substrate addition and acquisition were done as described before.

For measurement of human IgG, IgM and IgE antibodies from serum, bovine thyroglobulin (Sigma Aldrich) was coupled onto plates as described above. After washing, plates were blocked with chicken serum albumin (Sigma Aldrich). Diluted serum was added and incubated for 2 hours at RT. After washing, the biotinylated primary antibody specific for the indicated isotypes was incubated for 1 hour at RT. Detection was performed using streptavidin-HRP and acquisition was done as described before using TMB substrate.

### 2.17. Statistical analysis

For statistical analysis, a one-way ANOVA with Tukey’s multiple comparisons test was performed with GraphPad Prism 7 (GraphPad Software Inc.). A p value of p < 0.05 was considered statistically significant.

## 3. Results

### 3.1. The novel 27H8 monoclonal antibody specifically binds to α-Gal epitopes

In order to generate a monoclonal antibody specific for the α-Gal epitope determining structure Gal-α1,3-Gal that is equally able to bind to the naturally occurring α-Gal epitope Gal-α1,3-Gal-β1,4-GlcNAc, we immunized α-galactosyltransferase knockout mice (*Ggta1* KO) (26) with Gal-α1,3-Gal-β1,4-GlcNAc coupled to ovalbumin as carrier protein (α-Gal-OVA) according to the scheme depicted in **Figure 1A**. Splenic B cells were fused with the myeloma cell line P3X63-Ag8.653 and primary hybridoma supernatants were screened for IgG antibodies binding to Gal-α1,3-Gal-bovine serum albumin (α-Gal-DI-BSA) in a flow cytometric bead assay (**Figure 1A**). Screening for antibodies against Gal-α1,3-Gal coupled to a different carrier protein than used for immunization minimized the risk of pulling out antibody clones specific to the immunization molecule OVA. To further diversify immunization and screening molecule and avoid off-target (linker) specific antibodies, different linker lengths were selected with a 14-C-atom linker for the immunization molecule α-Gal-OVA and a 3-C-atom-linker for the screening molecule α-Gal-DI-BSA. Overall, 1536 supernatants from 4 immunized mice were screened and only two primary hybridoma supernatants (25G8 and 27H8) showed binding to α-Gal-DI-BSA. The determined isotype in the 25G8 primary hybridoma supernatant was IgG2b kappa, that of 27H8 IgG1 kappa. In a secondary screen, α-Gal-conjugated glycoproteins and respective control proteins without α-Gal were spotted on a membrane (dot blot) and incubated with either rat anti-mouse IgG1 (for 27H8) or rat anti-mouse IgG2b (for 25G8) and detected with anti-rat antibodies (**Figure 1B**). Both 25G8 and 27H8 primary hybridoma supernatants bound to α-Gal-conjugated mouse serum albumin (MSA), α-Gal-OVA and the disaccharide and trisaccharide α-Gal epitopes conjugated to BSA (α-Gal-DI-BSA/α-Gal-TRI-BSA). While 25G8 also strongly detected the carrier molecule OVA that was used for immunization, 27H8 showed only minimal binding to OVA (**Figure 1B**, upper two rows). Both primary hybridoma supernatants bound to glycolipids isolated from rabbit erythrocyte membranes rich in α-Gal (30). Binding was prevented by cleaving the carbohydrate from the lipid through pre-incubation with endoglycoceramidase I (EGCaseI) (**Figure 1B**), an enzyme hydrolysing the β-glycosidic covalent link between oligosaccharide and ceramide. In a second screening assay using a wet membrane and horseradish peroxidase (HRP)-labeled secondary antibodies, binding of 25G8 to OVA was still visible while binding of 27H8 was not detectable at all (**Figure 1C**). Therefore, 27H8 hybridoma cells were chosen for subcloning by limiting dilution to generate a stable monoclonal hybridoma cell line. Antibodies were purified from monoclonal 27H8 supernatant with protein A and both, supernatant and purified 27H8 antibody were validated alongside in a secondary dot blot screening with direct detection using an alkaline phosphatase (AP)-conjugated anti-mouse IgG antibody (**Figure 1D**). Both, the supernatant and purified 27H8 antibody showed a highly specific binding to all tested α-Gal carrying glycoproteins and -lipids but did not show any binding to OVA (**Figure 1D**). Thus, the initially observed weak binding of the primary 27H8 supernatant to OVA (**Figure 1B**) was most likely caused by a second hybridoma clone growing in the same well as 27H8, as in the first screening round monoclonality cannot be assumed. Next, the purified 27H8 antibody was conjugated to biotin. Successful biotinylation was validated in an enzyme-linked immuno assay (ELISA) by coating the biotinylated 27H8 as well as a biotinylated control antibody on plates followed by detection with streptavidin conjugated to HRP. The antibody 27H8 could be labeled with a similar efficiency as the control antibody (**Figure 1E**). The biotinylated 27H8 antibody in combination with Extravidin-AP showed a highly specific α-Gal detection without any detectable background staining to carrier molecules devoid of α-Gal (**Figure 1F**). In summary, the newly generated 27H8 monoclonal antibody binds to both the di- and trisaccharide epitope of α-Gal irrespective of its conjugation to proteins or lipids, it can be easily purified by protein A chromatography and can be labeled with biotin for enhanced detection and applicability.

### 3.2. 27H8 monoclonal antibody detects α-Gal epitopes of natural origin and offers a wide range of possible applications

To verify the specificity, the 27H8 monoclonal antibody was compared to *Bandereia simplifolica* isolectin B_4_ (BSI-B_4_) and to the monoclonal IgM antibody M86, which are both widely used to detect the α-Gal epitope (2, 18, 24). BSI-B_4_ is specific for terminal α-galactose oligosaccharides (35) and therefore recognizes also the blood group B antigen, which differs from the α-Gal epitope only in the addition of one fucose residue and is thus structurally very similar (36, 37). To assess whether 27H8 also binds to the blood group B antigen we blotted lysates of whole blood from a type B donor on a membrane and applied the antibodies 27H8 and M86 or biotinylated BSI-B_4_ for detection. While BSI-B_4_ bound to the blood type B specimen as expected, neither 27H8 or M86 did (**Figure 2A**). Next, we investigated whether 27H8 also binds to natural α-Gal epitopes. As pig kidney is naturally rich in α-Gal (38, 39) and reactions in α-Gal allergic patients are severe after ingestion (9), we tested if 27H8 recognizes α-Gal in pig kidney lysates in a dot blot assay. 27H8 binding to wildtype (WT) pig kidney lysate was observed with strong staining intensity (**Figure 2B** left panel). Control staining with the secondary anti-mouse IgG-AP antibody gave a faint signal on WT pig kidney lysate as well as on *GGTA1* KO cells lysates without or after 27H8 staining. However, no cross-reactivity of the secondary antibody was observed in WT pig kidney tissue lysates digested with α-galactosidase, an enzyme that cleaves off terminal α-galactose (40), indicating a relevance of galactose glycosylation for background staining by the secondary antibody. To avoid this background staining, we tested 27H8 on lysates from cultured pig kidney cells devoid of pig immunoglobulins. Here, background staining was not observed for anti-mouse IgG-AP on lysates from cultured cells and 27H8 bound exclusively to WT cultured pig kidney cells but not to *GGTA1* KO cultured pig kidney cells (**Figure 2B** middle panel). This result suggests that the secondary antibody used for detection still recognizes pig IgG antibodies present in whole kidney lysate despite anti-mouse-IgG-AP being highly cross-absorbed against immunoglobulins from various species. Additionally, we tested 27H8 on purified aminopeptidase N (APN) from HEK cells either expressing the α-1,3-galactosyltransferase or not. 27H8 only bound to α-Gal-APN and not to APN (**Figure 2B** right), further verifying its specificity to the α-Gal epitope. Importantly, 27H8 also recognizes bovine thyroglobulin – a protein used for α-Gal specific IgE antibody detection assays for red meat allergy patients [ImmunoCAP, Thermo Fisher Scientific, also described in (41)] (**Figure 2B** right). Specific binding of 27H8 to WT but not to *GGTA1* KO pig kidney was also observed on tissue slides using a monoclonal secondary antibody in immunohistochemistry (**Figure 2C**). 27H8 bound to the same cellular structures as M86 (**Figure 2C** upper left), such as binding to cells of the nephron’s tubular system but not to the glomerulus. Flow cytometry analysis of HEK cells expressing α-Gal-APN and APN confirmed specificity of 27H8 to natural α-Gal epitopes and highlights the broad range of applications of this antibody for detection of α-Gal epitopes in dot blot, histology and flow cytometry (**Figure 2D**). We therefore conclude that the 27H8 monoclonal antibody is highly specific for the α-Gal epitope in natural settings, does not bind the blood type B antigen and offers a wide range of possibilities for application

### 3.3. 27H8 monoclonal antibody binds with high affinity to α-Gal epitopes and competes with M86 for recognition

**Figure 3:**
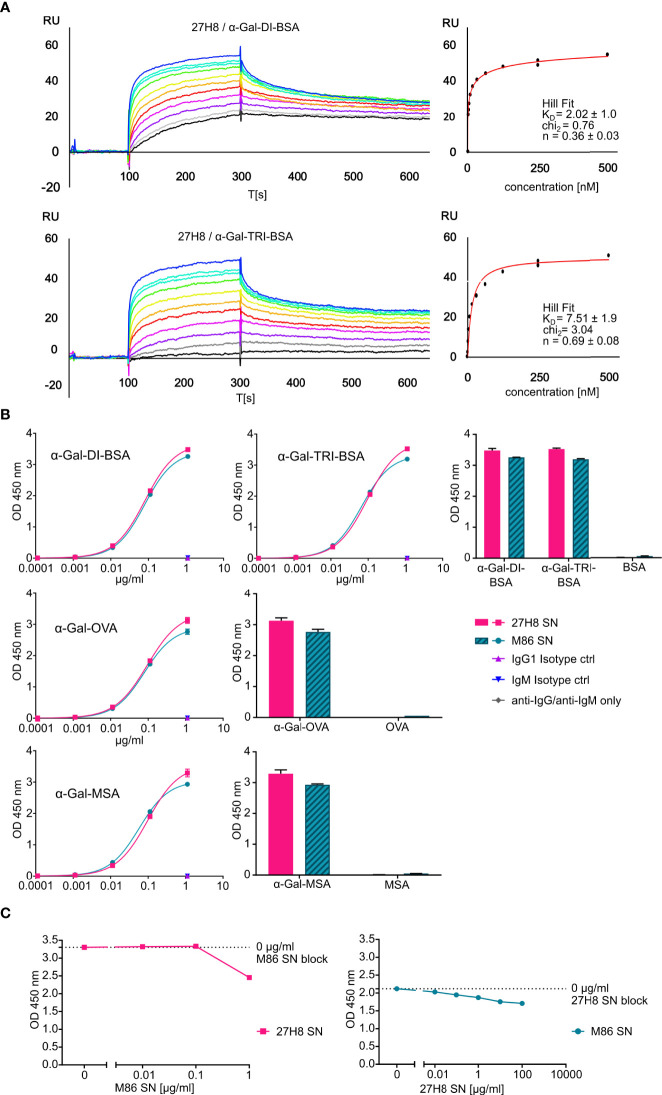
High affinity recognition of synthetic α-Gal epitopes. **(A)** Graphs show binding of synthetic α-Gal ligands to the coupled purified 27H8 antibody *via* surface plasmon resonance (SPR). Representative sensorgrams are displayed for 27H8/α-Gal-DI-BSA (upper panel) and 27H8/α-Gal-TRI-BSA (lower panel). For both pairs calculated equilibrium dissociation rate (K_D_), error as standard deviation from three independent experiments, the chi^2^ value for the curve fit and the Hill coefficient n are shown (right panel). Concentration series color code: black: 0.98 nM, gray: 1.95 nM, violet: 3.9 nM, magenta: 7.8 nM, red: 15.6 nM, orange: 31.2 nM, yellow: 62.5 nM, green: 125 nM, cyan: 250 nM, blue: 500 nM. RU: response units, T[s]: time in seconds. **(B)** Titration of 27H8 subcloned hybridoma SN and M86 hybridoma SN in ELISA on α-Gal conjugated glycoproteins (left) and direct comparison of glycoprotein and respective protein devoid of α-Gal at the highest concentration of 27H8 SN or M86 SN (1.12 µg/ml) (right). **(C)** Epitope blocking of α-Gal-MSA coated to ELISA-plates: M86 SN block followed by 27H8 SN and anti-IgG1 detection (left panel) or 27H8 SN block by M86 SN and anti-IgM detection (right panel). **(B, C)** Concentration values (x-axis) are plotted in logarithmic scale; antibody binding is shown as OD 450 nm (y-axis).

After determining the specificity and applicability of 27H8, we aimed to evaluate binding affinities of 27H8 antibody for the di- and trisaccharide α-Gal epitopes in a quantitative manner, and performed Surface Plasmon Resonance (SPR) analyses with both α-Gal-DI-BSA and α-Gal-TRI-BSA molecules (**Figure 3A**). Both analytes bound in a nanomolar concentration range to the coupled 27H8 antibody. The mean dissociation constant (K_D_) was slightly higher for α-Gal-TRI-BSA (7.51 ± 1.9) than for α-Gal-DI-BSA (2.02 ± 1.0), indicating a higher affinity of 27H8 for the disaccharide than the trisaccharide epitope. However, this might be partly explained by 35 sugar residues being attached to one molecule BSA for the α-Gal-DI-BSA analyte, while α-Gal-TRI-BSA consists of 33 sugar residues on average per protein. The Hill coefficients for the fitted binding curves of both analytes is smaller than one (n < 1), indicating negative cooperativity between the binding sites on 27H8 antibody (**Figure 3A**). Negative cooperativity suggests that the first binding analyte (α-Gal-DI-BSA or α-Gal-TRI-BSA) decreases the rate of subsequent analyte binding. As a full-length IgG antibody has two identical antigen-binding sites and due to the size of the BSA conjugated analytes (66kDa + 33 or 35 sugar residues), we assume that binding of one α-Gal-DI/TRI-BSA molecule to the first binding site on the 27H8 antibody may partially block the access of the second α-Gal-DI/TRI-BSA molecule to the second antigen-binding site as a result of steric hindrance. We next sought to compare the 27H8 antibody to M86, the most widely used monoclonal antibody specific for the α-Gal epitope developed by Galili *et al.* (24). This IgM antibody is commonly available as a hybridoma supernatant but for a direct affinity comparison both antibodies are ideally used in a purified format. However, while 27H8 antibody can easily be purified with protein A (**Figure 1D**), we were unable to purify M86 antibody with commonly used purification reagents such as recombinant protein L (Cytiva Capto™ L, Thermo Fisher Scientific, data not shown). Thus, we compared the hybridoma supernatants of 27H8 and M86 regarding their respective binding to α-Gal conjugated glycoproteins in an ELISA (**Figure 3B**). In order to titrate both antibodies to equal concentrations, we determined the antibody amount in the supernatants by interpolating OD 450 nm values to a standard curve of IgG1 and IgM isotype controls by a standard immunoglobulin isotype ELISA. To analyze the values in the linear range of the standard curve and dynamic range of the assay, 27H8 supernatant and M86 supernatant had to be diluted to variable degrees, which decreases the accuracy of concentration measurements. Thus, the concentrations of immunoglobulins in 27H8 supernatant and M86 supernatant are estimates. Additionally, antibody concentration in the 27H8 supernatant stock was approximately 100 times higher than in the M86 supernatant (27H8 supernatant: ~116.69 µg/ml; M86 supernatant: ~1.12 µg/ml) (see Material and Methods). When comparing 27H8 supernatant and M86 supernatant titration curves on α-Gal-DI-BSA and α-Gal-TRI-BSA coated to ELISA-plates, we observed that both antibodies bind the di- and trisaccharide epitopes of α-Gal with a similar avidity (**Figure 3B** upper panel). This similar binding property was also observed on α-Gal-OVA- (**Figure 3B** middle panel) and α-Gal-MSA-coated plates (**Figure 3B** lower panel). Supernatant of subcloned monoclonal 27H8 hybridoma did not bind respective proteins devoid of α-Gal, such as BSA, MSA and, importantly, it did not bind OVA (**Figure 3B** right), in contrast to the weak binding of the primary hybridoma supernatant to OVA (**Figure 1B**). To further confirm that 27H8 and M86 recognize the α-Gal epitope in a similar manner we performed blocking assays in which the antibodies competed with each other for α-Gal binding (**Figure 3C**). α-Gal-MSA was coated onto ELISA-plates and incubated with increasing amounts of either 27H8 supernatant or M86 supernatant in a serial dilution to block the α-Gal epitope. The maximum concentration of M86 used for blocking was limited to 1 µg/ml due to the low stock concentration, while 27H8 supernatant was increased up to 100 µg/ml. Afterwards, the respective competing antibody was added (27H8 to M86 block and M86 to 27H8 block) and detected with specific anti-IgG1 or anti-IgM antibodies, respectively. 27H8 supernatant binding was blocked by M86 at concentrations higher than 0.1 µg/ml while 27H8 supernatant blocked M86 binding gradually even at lower amounts (starting from ~0.01 µg/ml). This discrepancy might be explained by the different isotypes (IgG1 vs IgM) and steric inhibition by IgM pentamers, but also confirms the high affinity of 27H8 monoclonal IgG1 antibody to the α-Gal epitope. Furthermore, we confirmed that 27H8 has a different variable domain sequence in the CDR regions compared to M86 which translates also in a different amino acid sequence and thus epitope recognition [(42) and data not shown]. In brief, the novel 27H8 antibody binds the α-Gal epitope comparable to M86 in ELISA and displays high affinity for its epitope.

### 3.4. *Staphylococcus aureus* does not express the α-Gal epitope

**Figure 4:**
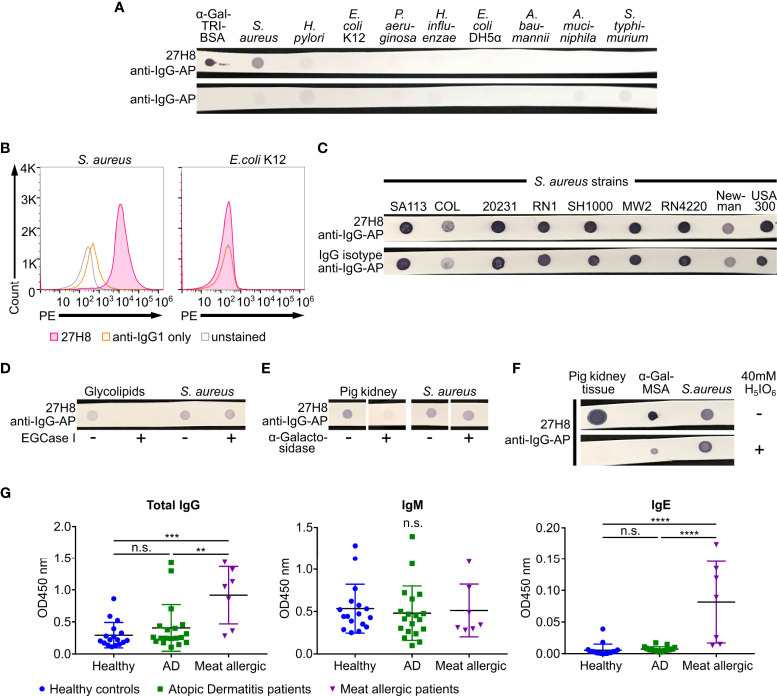
*Staphylococcus aureus* strains bind 27H8 independent of α-Gal expression. **(A)** Dot blots of the positive control α-Gal-TRI-BSA and lysed bacterial species *Staphylococcus aureus* (*S. aureus*, strain Mu50), Helicobacter pylori (*H. pylori*), *Escherichia coli (E. coli*, K12 and DH5α), *Pseudomonas aeruginosa (P. aeruginosa)*, *Haemophilus influenzea (H. influenzae*), *Acinetobacter baumannii (A. baumannii), Akkermansia muciniphila (A. muciniphila)* and *Salmonella typhimurium (S. typhimurium*). **(B)** Histograms of flow cytometric analysis of *S. aureus* strain 20231 and *E.coli* K12 stained with 27H8 and anti-IgG1-PE. **(C)** Multiple *S. aureus* strains stained with 27H8 and IgG isotype in dot blot. **(D)** Dot blot of glycolipids and *S. aureus* strain SH1000 digested or not with EGCase I as indicated. **(E)** Dot blot of pig kidney lysate and *S. aureus* strain SH1000 digested or not with α-Galactosidase as indicated. Uncropped blots depicted in **Supplementary Figure 1D**. **(F)** Pig kidney lysate, α-Gal-MSA and *S. aureus* strain 20231 blotted on membrane and either incubated with Periodic acid (H_5_IO_6_) or not. **(A, D–F)** Detection with 27H8 and anti-IgG-AP. **(A-F)** 27H8 was applied in the purified version. **(G)** ELISA of human IgG, IgM and IgE binding to thyroglobulin in serum samples from healthy controls, Atopic Dermatitis (AD) or red meat allergic patients. Each symbol represents an individual subject. Statistics: one-way ANOVA with Tukey’s multiple comparisons test, **p <0.01, ***p < 0.001, ****p < 0.0001, n.s.: not significant.

Intestinal bacteria have been hypothesized to induce anti-α-Gal immunoglobulins (IgM, IgG) in humans (16). Thus, we investigated whether 27H8 antibody binds to bacteria reported to express α-1,3-galactosyltransferase-like genes (KEGG orthology number KO3275 or KO3278) as described by Montassier *et al.* (20), such as *H. pylori* (J99), *H. influenzae* (Hi375), *S. typhimurium* (ATCC 14028)*, P. aeruginosa* (DSM 50071), *A. baumannii* (ATCC 17978) and *A. muciniphila* (ATCC BAA-835). Negative controls were selected according to literature, such as *E. coli* K12 (18). We further included *E. coli* DH5α and strains from the gram positive bacterium *S. aureus*, though it was reported that most α-Gal expressing bacteria were supposed to be gram-negative (20). Surprisingly, none of the tested bacterial lysates could be stained with the 27H8 antibody in a dot blot experiment, except *S. aureus* Mu50 and as positive control α-Gal-TRI-BSA (**Figure 4A**). The binding of 27H8 to *S. aureus* was not only observed in a dot blot but also by bacterial flow cytometry (**Figure 4B**). The fluorescence intensity increased in the secondary antibody only sample (anti-IgG1-PE) relative to the unstained control indicating a substantial background stain (**Figure 4B**, left panel). However, the first IgG1 isotype control we used (Southern Biotech) did not give the same fluorescence signal as 27H8 antibody when applying the same concentration (data not shown). We further observed that 27H8 binding to *S. aureus* is a shared pattern for multiple strains but if we applied a polyclonal IgG isotype control, the same staining intensity was observed as for 27H8 (**Figure 4C**). This result suggests that the binding of 27H8 to *S. aureus* strains is likely a common feature of IgG antibodies regardless of specificity and is not due to a specific binding to the α-Gal epitope present on this bacterium. To further demonstrate that *S. aureus* does indeed not express α-Gal, we cleaved and removed the α-Gal epitope by various approaches and examined 27H8 binding thereafter. First, we removed the α-Gal carrying oligosaccharide in the *S. aureus* sample by EGCase I digestion, but in contrast to control digestion of glycolipids, no signal was lost for *S. aureus* (**Figure 4D**). Furthermore, when comparing pig kidney tissue lysate and *S. aureus* lysate digested with α-Galactosidase, we observed a significant signal reduction for the mammalian sample, but not for the bacterial sample (**Figure 4E**). Finally, when the membrane of blotted samples of pig kidney tissue lysate, α-Gal-MSA and *S. aureus* lysate was pre-incubated with periodic acid, a treatment that destroys all carbohydrate determinants (43), the staining intensity of 27H8 was lost or substantially reduced for pig kidney and α-Gal MSA, but not for the *S. aureus* sample (**Figure 4F**). Thus, the 27H8 antibody binds to a structure in *S. aureus* that is not part of an oligosaccharide connected to a sphingolipid, does not contain α-galactose residues and is not even a carbohydrate. Most probably, 27H8 binds to protein A, as already implied for human polyclonal anti-α-Gal antibodies binding to *S. aureus* human isolates (17). In line with this result, we could not detect any staining of *S. aureus* samples with M86 in a dot blot as IgM antibodies are typically not bound by protein A (data not shown). Furthermore, and in contrast to meat allergic patients, we did not observe enhanced IgG or IgE titers against bovine thyroglobulin [a molecule routinely used to detect anti-α-Gal antibodies in patient serum samples (41)] in our selection of atopic dermatitis patients (**Figure 4G**). We selected this patient group as atopic dermatitis patients are usually strongly colonized by *S. aureus* (44). Serum IgM titers against thyroglobulin were unchanged between the groups. Altogether, these data strongly indicate that *S. aureus* binds *via* protein A to the constant part of the 27H8 antibody and does not express α-Gal itself.

### 3.5. 27H8 and M86 antibodies do not bind to *E. coli* O86:B7 nor to other members of the intestinal microbiota

**Figure 5:**
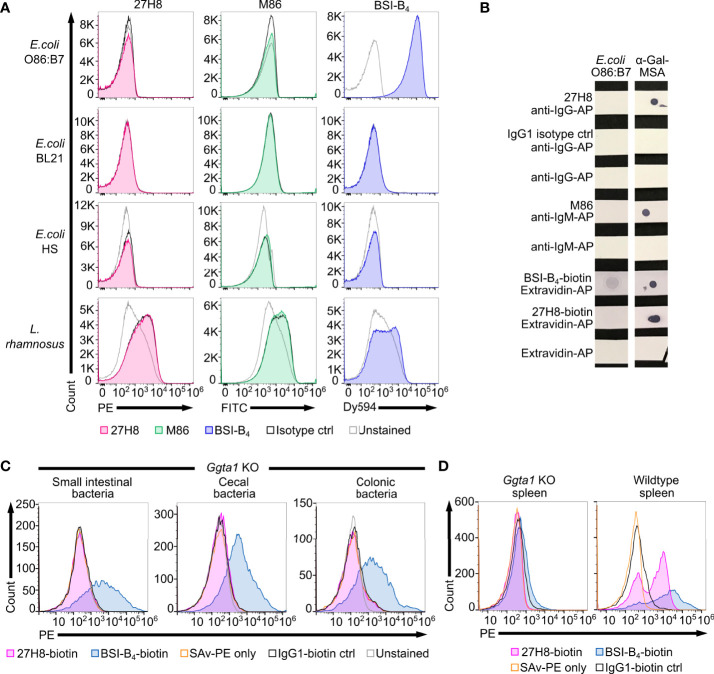
No binding of 27H8 and M86 to intestinal bacteria in contrast to BSI-B_4._ **(A)** Histograms of flow cytometric analysis of cultured bacterial strains stained with 27H8, M86 and BSI-B_4_ and respective isotype controls (IgG1 and IgM). Strains: *E coli* O86:B7, *E.coli* BL21, *E coli* Human Species (HS) and *Lactobacillus rhamnosus* (*L. rhamnosus*). **(B)** Dot blot stain of lysed *E coli* O86:B7 and α-Gal-MSA as positive control. Uncropped blots depicted in **Supplementary Figure 1E**. **(C)** Representative histogram blots of flow cytometric analysis of intestinal content (derived from small intestine, cecum and colon) from *Ggta1* KO mice (n=3) stained with biotinylated 27H8 and BSI-B_4_, pre-gated for SYBR green positive bacteria (**Supplementary Figure 2A**). **(D)** Live splenocytes of *Ggta1* KO and WT mouse (**Supplementary Figure 2B**) stained with biotinylated 27H8 and BSI-B_4_. **(C, D)** Control staining with IgG1-biotin isotype and streptavidin-PE (SAv-PE) only.

As we did not observe any binding of 27H8 antibody to lysates of cultivated bacteria in a dot blot (**Figure 4A**), we next sought to test for binding of 27H8 to *E. coli* O86:B7. This strain was reported to express α-Gal detected by BSI-B_4_ in multiple studies (18, 19, 45), and is frequently used as a positive control as it has also been shown to induce anti-α-Gal antibodies in *Ggta1* KO mice after oral inoculation (22). Surprisingly, neither 27H8 nor M86 antibody bound to *E. coli* O86:B7 while BSI-B_4_ strongly stained this bacterial strain (**Figure 5A**). This was specific for *E. coli* O86:B7 because the negative control *E. coli* BL21, described in (46), was not stained by the lectin. We also tested further bacteria such as an *E. coli* strain isolated from human feces (*E. coli* HS) and *Lactobacillus rhamnosus* which showed minimal α-Gal positive staining by BSI-B_4_ in (19). We could not observe any specific binding of 27H8 or M86 to these two strains (**Figure 5A**). BSI-B_4_ did not bind to *E. coli* HS and showed a slight signal shift compared to the unstained control for *L. rhamnosus*. We could rule out technical errors of 27H8 applied in flow cytometry since α-Gal expressing HEK cells were indeed stained by this antibody using the same technique (**Figure 2D**). The binding of BSI-B_4_ to *E. coli* O86:B7 in contrast to 27H8 and M86 could also be observed in a dot blot using lysates of this strain (**Figure 5B**). As it has been suggested that the induction of anti-α-Gal antibodies and also immunological tolerance towards this epitope might be driven by the intestinal microbiota (16), we wondered whether 27H8 binds to intestinal bacteria at all. Therefore, we incubated bacteria from the intestinal compartments of *Ggta1* KO mice with 27H8 for antibody binding and performed bacterial flow cytometry. To avoid anti-mouse secondary antibody attaching to murine immunoglobulins contained in the samples, we applied the biotinylated version of 27H8 and BSI-B_4_ as control. While BSI-B_4_-biotin bound to a large number of intestinal bacteria from the small intestine, cecum and colon, this was not visible for the biotinylated 27H8 antibody as there was no signal shift observable exceeding the streptavidin-PE (SAv-PE) only control or the biotinylated IgG1 control (**Figure 5C**). To confirm that also the biotinylated version of 27H8 binds to α-Gal in flow cytometry we applied the same technical setup as for the intestinal bacteria to splenocytes from *Ggta1* KO and WT mice. While biotinylated 27H8 bound to splenocytes from WT mice, no binding to splenocytes from *Ggta1* KO mice was detectable (**Figure 5D**). Altogether, we conclude that neither of the two α-Gal binding monoclonal antibodies 27H8 or M86 bind to structures on the bacterial surface or in lysates while the lectin BSI-B_4_ indeed binds to bacterial epitopes most likely in a non-α-Gal epitope specific manner.

## 4. Discussion

Humans naturally display antibodies reactive to Gal-α1,3-Gal, the determining structure of the α-Gal epitope, that exhibit a broad range of pathogen reactivity and can also bind to non-α-Gal epitopes such as human blood group B, A and 0 (17). Different approaches have been used to purify such polyclonal and potentially cross-reactive anti-α-Gal antibodies (16, 47), yet they may also bind to non-Gal-α1,3-Gal expressing organisms (17). Previously, it has been hypothesized that anti-α-Gal antibodies are induced by the intestinal microbiota as *Escherichia coli*, *Klebsiella* and *Salmonella* strains can bind to polyclonal anti-α-Gal antibodies purified with Gal-α1,3-Gal-β1,4-Glc (16). For instance, oral inoculation of *Ggta1* KO mice with the *E. coli* strain O86:B7 has been shown to elicit enhanced anti-α-Gal titers (22). However, only the expression of α-Gal-like structures has been demonstrated for this strain to date as an additional fucose residue is attached to Gal-α1,3-Gal (48). To determine if bacteria express the α-Gal epitope defined as Gal-α1,3-Gal or Gal-α1,3-Gal-β1,4-GlcNAc without further residues attached to the second last galactose, the use of polyclonal, cross-reactive human anti-α-Gal antibodies might therefore lead to false positive results. Similarly, the lectins BSI-B_4_ and MOA, binding also to α-Gal-like structures such as the blood group B antigen, do not exclusively recognize the α-Gal epitope. However, both polyclonal human anti-α-Gal antibodies and lectins have been used to demonstrate α-Gal epitope expression by bacteria and the microbiota in the past (18, 20).

Monoclonal antibodies allow a more precise epitope recognition after excluding cross-specificity as presented in this study. Here, we describe a novel, monoclonal IgG1 antibody called 27H8 which binds both the di- and trisaccharide α-Gal epitope with high affinity but does not display cross-reactivity to the blood group B antigen. Throughout the study, we demonstrate that the 27H8 monoclonal antibody binds to the same α-Gal containing structures as the most commonly used monoclonal IgM antibody called M86. We also show that M86 binds to both the di- and trisaccharide α-Gal epitope (Gal-α1,3-Gal or Gal-α1,3-Gal-β1,4-GlcNAc). The M86 antibody has been developed by Galili *et al.* in a similar approach by immunizing *Ggta1* KO mice with α-Gal rich rabbit red blood cells (24) in contrast to synthetic α-Gal-OVA used in our study. Since SPR-affinity studies indicate that the K_D_s of the variable regions of the pentamer IgM antibody M86 genetically engineered to scFv-IgE antibodies (49) are higher than 27H8, we assume 27H8 variable regions bind to α-Gal at a higher affinity than M86. We used a broad screening approach utilizing cell lysates and purified α-Gal-rich proteins and lipids, and demonstrate specificity of 27H8 to α-Gal *via* enzymatic digestion, the use of *Ggta1* KO mice and pigs as well as transgenic expression of α1,3-galactosyltransferase in HEK cells. The 27H8 antibody recognizes α-Gal-conjugated proteins or natural α-Gal-rich compounds and glycolipids and is applicable in dot blot, immunohistochemistry, ELISA and flow cytometry, demonstrating robustness in its α-Gal epitope recognition. As the 27H8 antibody does not bind to the blood group B antigen, we conclude that further residues on the core galactose limits antibody binding to α-Gal. Additionally, this antibody displays unique features and advantages when compared to M86 as 27H8 is easily purifiable and can thus be directly labeled with fluorophores or enzymes for example to design improved ELISA systems.

As one first application, we used the 27H8 antibody to test the hypothesis if intestinal bacteria are a major source of α-Gal possibly involved in the sensitization of the immune system. Therefore, we applied the 27H8 antibody to lysates of bacteria hypothesized to be α-Gal expressing organisms *via* their expression of α1,3-galactosyltransferase-like genes (20). Strikingly, we did not detect any binding of 27H8 to *H. pylori*, *H. influenzae*, *S. typhimurium*, *P. aeruginosa, A. baumannii* and *A. muciniphila*. This lack of binding could also be demonstrated for *E. coli* O86:B7, another human *E. coli* isolate, *L. rhamnosus* and more generally for the majority of murine intestinal bacteria isolated from *Ggta1* KO mice. As this is a negative result, we cannot exclude that the 27H8 antibody binds to bacteria not tested in this setup or under different experimental conditions. However, as 27H8 also failed to stain murine intestinal bacteria derived from a host devoid of α-Gal, we propose that intestinal bacteria are generally devoid of the native α-Gal epitope. Similarly, the use of M86 for bacterial α-Gal epitope detection has not been shown convincingly and many studies relied on the use of lectins for this purpose (18–20). In our setting, we could equally not observe any binding of M86 when applied by flow cytometry or to lysates of cultured bacteria. We therefore conclude that either Gal-α1,3-Gal is not present on the tested bacteria and the intestinal microbiome, or it must be part of a more complex structure that shields antibody recognition by high-affine 27H8 and also M86. Consequently, the defining α-Gal epitope structure Gal-α1,3-Gal without further residues attached may not be expressed by bacteria at all. Another possibility might be that the α-Gal epitope is only revealed after processing the bacterial oligosaccharide structures by the host. Therefore, we strongly recommend to carefully differentiate between the expression of the actual α-Gal epitope, namely Gal-α1,3-Gal, and the expression of α-Gal-like glycans, e.g., α-Galactose residues connected *via* 1,3 linkages to other saccharides or further residues connected to the core galactose to avoid incorrect assumptions. In contrast to non-primate mammals and certain parasites, intestinal bacteria have been shown to express only α-Gal-like oligosaccharide structures (50) that may elicit initially low affine anti-α-Gal IgM antibodies. According to this scenario, a second yet to be discovered genuine α-Gal epitope source then triggers affinity maturation and IgG antibody production from this pool of B cells.

Additional methods to elucidate glycan structures on microbes may be nucleic magnetic resonance spectroscopy or reversed immunoglycomics as shown for *Leishmania major* (51). Moreover, we further encourage to carefully control experiments related to the destruction of the α-Gal epitope by using enzymatic digestion or periodate, as also described for anti-α-Gal IgE antibodies (39), to show the specificity of anti-α-Gal epitope recognition. We realized a binding of 27H8 antibody to *S. aureus* strains independent from the α-Gal epitope, an observation that was also made for human anti-α-Gal antibodies in another study. However, also in that study, these antibodies might have bound to protein A and not to the genuine α-Gal epitope (17).

In future studies, it will be interesting to apply 27H8 antibody to parasites suggested to express the actual α-Gal epitope, such as *Trypanosoma brucei* (52), *Ascaris lumbricoides* (53) and also *Plasmodium* species (18, 54) in order to investigate whether recognition of the α-Gal epitope is generally used by the immune system to recognize parasites. Additionally, 27H8 can be used to gain mechanistic insight into the ‘red meat allergy’ phenomenon mediated *via* tick bites (55), as compartmentalized α-Gal expression in tick species has been shown by an overlay staining of MOA and M86 (56). Lastly, xenotransplantation approaches of mammalian and in particular pig organs transplanted into human recipients heavily rely on the complete absence of the α-Gal epitope or the need to eradicate transplant reactive anti-α-Gal antibodies in the recipient prior to transplantation. The 27H8 antibody may be used to develop diagnostic tests and tools for α-Gal expression in diets and prior to organ transplantation and develop more sensitive sandwich ELISA tests to determine anti-α-Gal isotype levels in patients.

Altogether, we describe here a rigorously characterized and novel monoclonal IgG1 antibody that reliably recognizes the α-Gal epitope with high affinity and specificity. Using this novel tool, we propose to carefully re-evaluate bacterial α-Gal expression as a major epitope source and advocate for essential control stainings using several isotypes and enzymatic cleavage of the epitope to prove genuine α-Gal epitope expression in a given sample.

## Data availability statement

The original contributions presented in the study are included in the article/**Supplementary Material**. Further inquiries can be directed to the corresponding author.

## Ethics statement

The studies involving human participants were reviewed and approved by Ethical committee from the Technical University of Munich, School of Medicine, ethical vote numbers 419/18 S-KK and 5590/12. The patients/participants provided their written informed consent to participate in this study.

## Author contributions

LK and CO designed the study. LK performed and analyzed most experiments. AS, RF, AF performed immunization protocols and monoclonal antibody generation. RJ performed affinity measurements. KS helped with flow cytometric analyses of HEK cells and bacteria. KF, MD, NC contributed to screening experiments. MK, LD, MSD contributed to bacterial experiments. MH, SE, DN, CS-W, SB, CH, TB contributed to experimental work or gave critical input. LK and CO wrote the manuscript with input from coauthors. All authors contributed to the article and approved the submitted version.

## Funding

This work was supported by intramural funds from Helmholtz Munich and from the European Research Council (ERC Starting grant) project number 716718 to CO. The Ohnmacht lab is further supported by grants from the Deutsche Forschungsgemeinschaft (DFG) grant number OH 282/1-2 within FOR2599 to CO and project P07 within SFB1371 to CO. The Biedermann lab is supported by the DFG funded grants Project BI 969/12-1, the CRC1371 (Project P06), the CRC1335 (Project P17) and the RTG 2668. NC and CH were supported by a bilateral grant from the Luxembourg National Research Fund (FNR), project C17/BM/11656090 and the DFG.

## Acknowledgments

We thank Johanna Grosch, Sabrina Engel and Alessandro De Sciscio for technical assistance, Maximilian Schiener for providing screening samples, Ann-Marie Maier for blood donor acquisition and, including Anna-Lena Geiselhöringer, Amelie Köhler, Maria Szente-Pasztoi, Daphne Kolland, Christian Hoffmann, Jinlong Ru and Florian Wölbing for helpful discussions.

## Conflict of interest

MSD works as a consultant and an advisory board member at Theralution GmbH, Germany.

The remaining authors declare that the research was conducted in the absence of any commercial or financial relationships that could be construed as a potential conflict of interest.

## Publisher’s note

All claims expressed in this article are solely those of the authors and do not necessarily represent those of their affiliated organizations, or those of the publisher, the editors and the reviewers. Any product that may be evaluated in this article, or claim that may be made by its manufacturer, is not guaranteed or endorsed by the publisher.
