# Supplementary material for: A novel monoclonal IgG1 antibody specific for Galactose-alpha-1,3-galactose questions alpha-Gal epitope expression by bacteria
Source: Front Immunol. 2022 Aug 5;13:958952. doi: 10.3389/fimmu.2022.958952 (PMC9391071; doi:10.3389/fimmu.2022.958952)
Supplement: Supplementary file 1 [file DataSheet_1.docx]

**
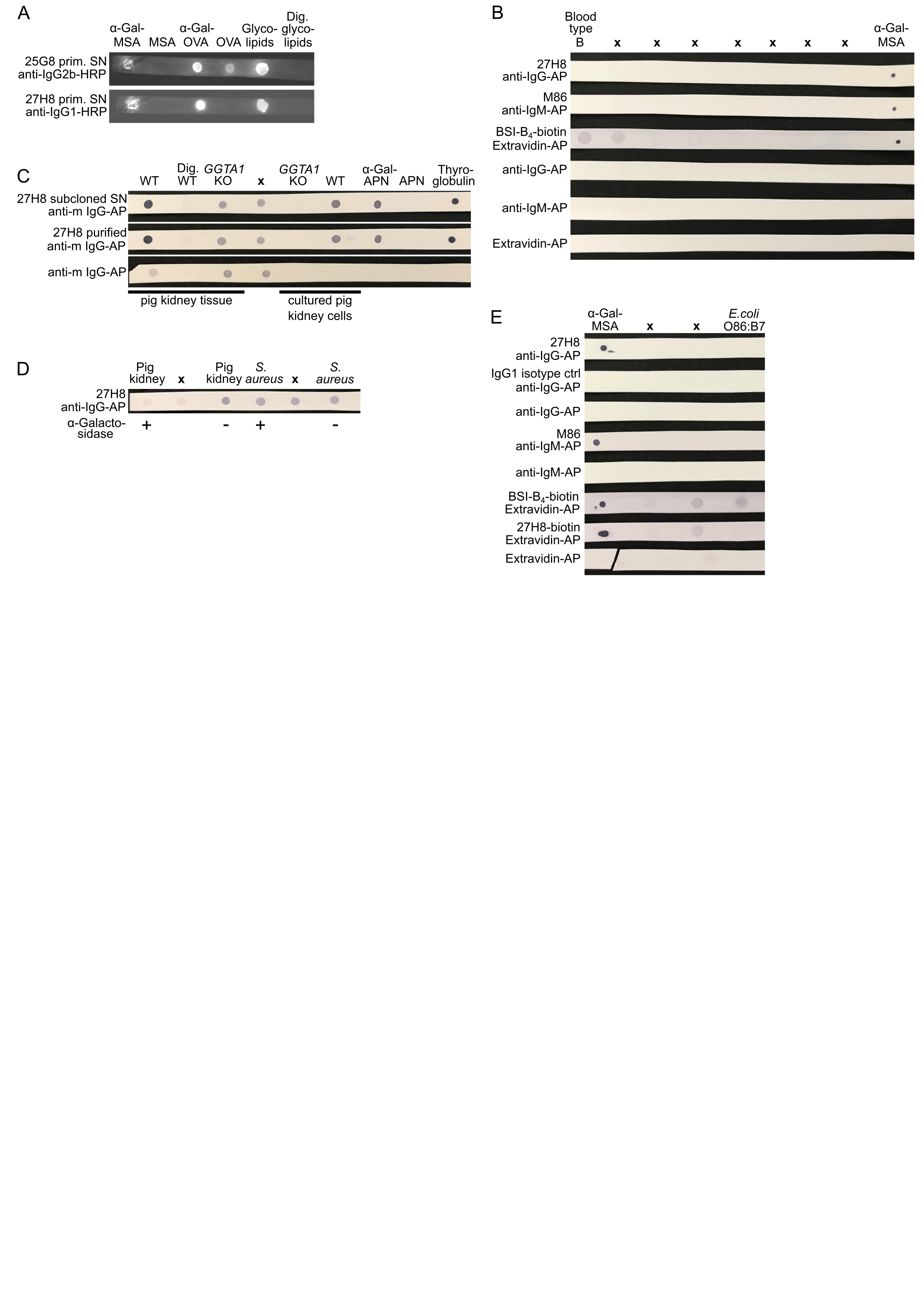
Supplementary Figure 1.** Uncropped dot blots appearing in **Figures 1-5**. **(A)** Uncropped dot blot of **Figure 1C**, **(B)** of **Figure 2A**, **(C)** of **Figure 2B**, **(D)** of **Figure 4E**, **(E)** of **Figure 5B**. Samples not mentioned in the manuscript due to irrelevance and which have been used only to test conditions are labeled with **x**.

**
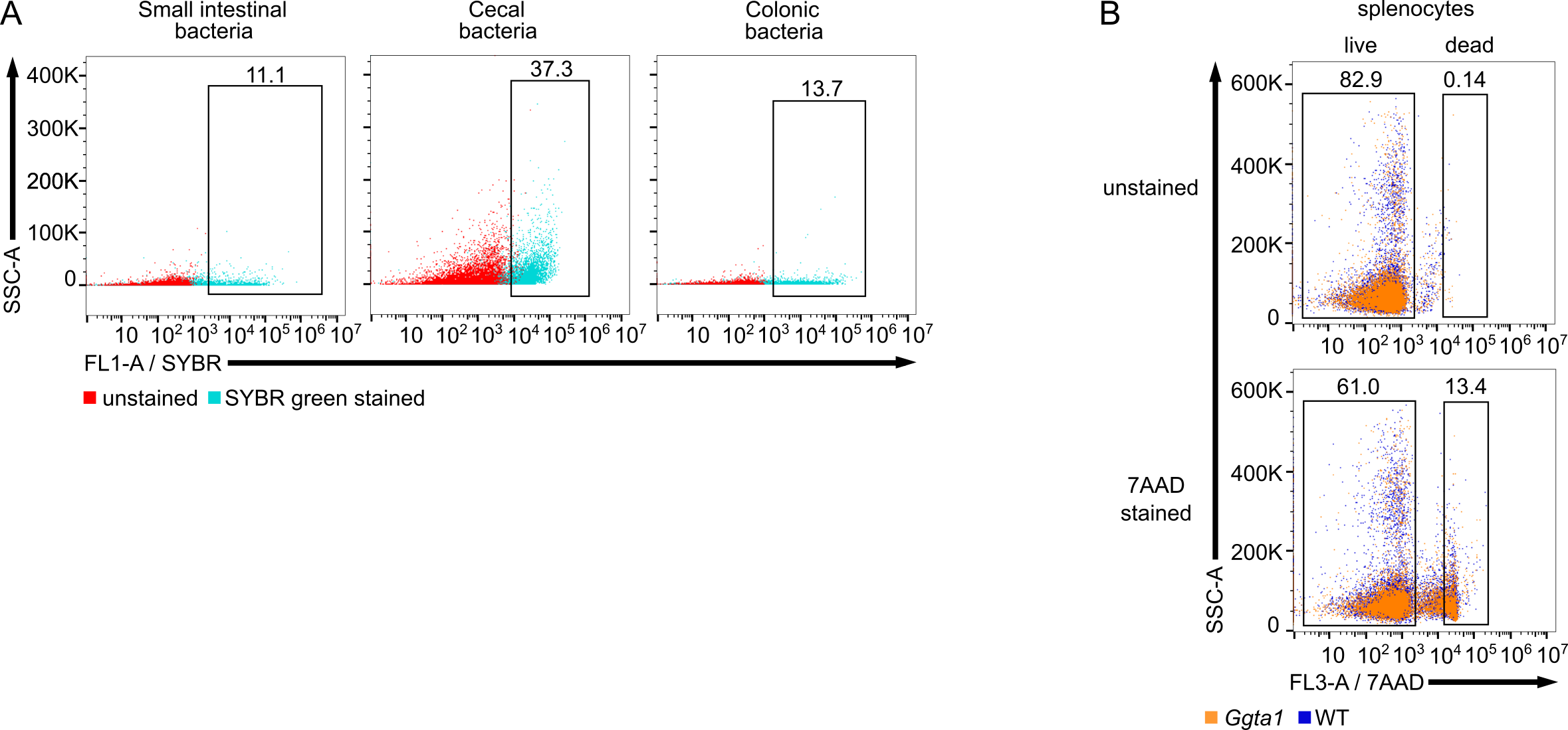
**

**Supplementary Figure 2.** Representative flow cytometry plots displaying pre-gating for bacterial and live cell identification for plots shown in **Figure 5 C-D**. **(A)** Identification of bacteria via SYBR green positive staining of intestinal contents of small intestine, cecum and colon **(B)** Identification of live murine splenocytes via staining with 7AAD.
